# Pulmonary thromboembolism in Glanzmann Thrombasthenia: a case report and systematic literature review

**DOI:** 10.1007/s00277-026-06732-8

**Published:** 2026-01-27

**Authors:** Tayebe Mohammad Alizade, Reihaneh Karimi, Hossein Kazemizadeh, Niloofar Khoshnam Rad

**Affiliations:** 1 Thoracic Research Center, Imam Khomeini Hospital Complex, Tehran University of Medical Sciences, Tehran, Iran; 2 Department of Internal Medicine, School of Medicine, Tehran University of Medical Sciences, Tehran, Iran

**Keywords:** Pulmonary embolism, Glanzmann thrombasthenia, Direct oral anticoagulants, Anticoagulants, Postoperative complications

## Abstract

Glanzmann Thrombasthenia (GT) is a congenital platelet disorder characterized by a life-long bleeding tendency, historically considered protective against thrombosis. This report describes a rare case of pulmonary embolism (PE) in a patient with GT, challenging this assumption and highlighting a critical management paradox. A 55-year-old woman with GT underwent elective cervical discectomy. Her perioperative hemostatic regimen included a single prophylactic dose of recombinant Factor VIIa (90 µg/kg), platelet transfusions (preoperative and daily for 3 days), and tranexamic acid. Two months postoperatively, she presented with hemoptysis. CT pulmonary angiography confirmed bilateral segmental and subsegmental PE with pulmonary infarctions. Anticoagulation with apixaban was initiated, leading to symptom resolution and thrombus regression. Therapy was discontinued after four months following a spontaneous hemarthrosis, which was managed successfully. Provoked PE can occur in GT patients following major surgery and hemostatic support. Apixaban was effective for short-term treatment, but the subsequent bleeding complication highlights the narrow therapeutic window and the need to limit anticoagulation duration in this population.

**Supplementary Information:**

The online version contains supplementary material available at 10.1007/s00277-026-06732-8.

## Background

Glanzmann Thrombasthenia (GT) is a rare autosomal recessive platelet disorder caused by a deficiency or dysfunction of the glycoprotein IIb/IIIa complex (αIIbβ3 integrin), which is essential for platelet aggregation. Patients consequently experience a lifelong bleeding tendency characterized by mucocutaneous hemorrhages, menorrhagia, and bleeding following trauma or surgery. Historically, GT has been considered protective against arterial and venous thrombosis due to this inherent hypocoagulability [1]. This perspective has been critically reviewed in the context of modern hemostatic management and the complex biology of β3 integrins, which can themselves introduce or modulate thrombotic risk [2].

Emerging evidence indicates thrombotic complications can occur in GT, particularly with exogenous prothrombotic stimuli (e.g., surgery, immobilization, factor concentrates) [3, 4]. The interplay between platelet dysfunction and acquired thrombotic risks remains poorly understood. Recent cases describe venous thromboembolism (VTE) in GT, often linked to recombinant Factor VIIa (rFVIIa) or acquired hypercoagulable states [3, 5]. While factor replacement restores hemostasis temporarily, overuse may promote thrombin generation. Platelet transfusions, though essential for bleeding control, can trigger hypercoagulable pathways [6].

We present a case of PE in a patient with GT, followed by a systematic review of the literature. This report aims to highlight the hemorrhage-thrombosis paradox and discuss the challenges of antithrombotic management in congenital bleeding disorders.

## Methods: literature review

The systematic review was conducted in accordance with PRISMA guidelines. A systematic search of PubMed and Scopus was conducted from inception until August 2025. The complete search strategy for all databases and a PRISMA flow diagram detailing the study selection process are provided in Supplementary Material 1. Two reviewers independently screened titles and abstracts, followed by full-text assessment of potentially eligible articles. Articles were included if they were published in English and provided sufficient clinical detail on the management of VTE in a patient with a confirmed or clinically robust diagnosis of GT. Case reports, case series, and letters to the editor with sufficient clinical data were eligible. Reviews and articles without original patient data were excluded. Data on patient demographics, VTE event type, provoking risk factors, management strategies, and outcomes including anticoagulation duration were extracted into a standardized table. Case reports and series meeting these criteria were analyzed for presentation, risk factors, management strategies, and outcomes. The risk of bias in included case reports was assessed using the JBI Critical Appraisal Checklist for Case Reports.

## Case presentation

A 55-year-old woman with a well-established diagnosis of GT and stable hypothyroidism presented with a 5-day history of non-massive hemoptysis. The diagnosis of GT had been made in childhood based on a characteristic clinical bleeding history (including lifelong epistaxis, menorrhagia, and easy bruising) and confirmed by standard light transmission aggregometry (LTA). LTA showed absent platelet aggregation in response to ADP, epinephrine, and collagen, with normal aggregation to ristocetin. Flow cytometric analysis had further confirmed a severe deficiency of platelet surface GPIIb (CD41: <2%) and GPIIIa (CD61: <2%). Genetic sequencing had not been performed due to limited resource availability; however, the combined clinical and phenotypic findings are definitive for GT.

Two months prior to presentation, she underwent an elective anterior cervical discectomy and fusion. A review of the hematology consultation and anesthesiology records revealed the following perioperative hemostatic plan: a single prophylactic dose of recombinant Factor VIIa (rFVIIa) at 90 µg/kg (4.8 mg) was administered preoperatively. Further doses of rFVIIa were reserved for rescue therapy in case of significant intraoperative bleeding; however, no such bleeding occurred, and no additional rFVIIa was administered. Prophylactic platelet transfusions (2 units) were given preoperatively and then daily for the following 3 days. A single intravenous dose of 1 gram, administered prophylactically during the perioperative period. Her hypothyroidism was well-controlled on levothyroxine, and there was no personal or family history of thrombosis.

On presentation, she was hemodynamically stable and afebrile. Physical examination revealed only mild pallor and a well-healed anterior cervical surgical scar. Laboratory investigations are summarized in Table 1.

**Table 1:** Baseline laboratory investigations

Parameter (Unit)	Value	Reference Range
White Blood Cell Count (×10³/µL)	4.7	4.5–11.0
Hemoglobin (g/dL)	11.1	12–16
Platelet Count (×10³/µL)	294	150–450
Prothrombin Time (sec)	14.3	11–15
Partial Thromboplastin Time (sec)	33	25–40
International Normalized Ratio	1	1–1.4
Fibrinogen (mg/dL)	**285**	**200–400**
D-dimer (ng/mL)	**1250**	***< 500***
Creatinine (mg/dL)	0.8	0.7–1.4
Aspartate Aminotransferase (U/L)	36	< 31
Alanine Aminotransferase (U/L)	26	< 31
Alkaline Phosphatase (U/L)	260	70–460
Troponin (ng/mL)	< 0.02	≤ 0.29
B-type Natriuretic Peptide (pg/mL)	11.7	≤ 160

**Fig. 1:**
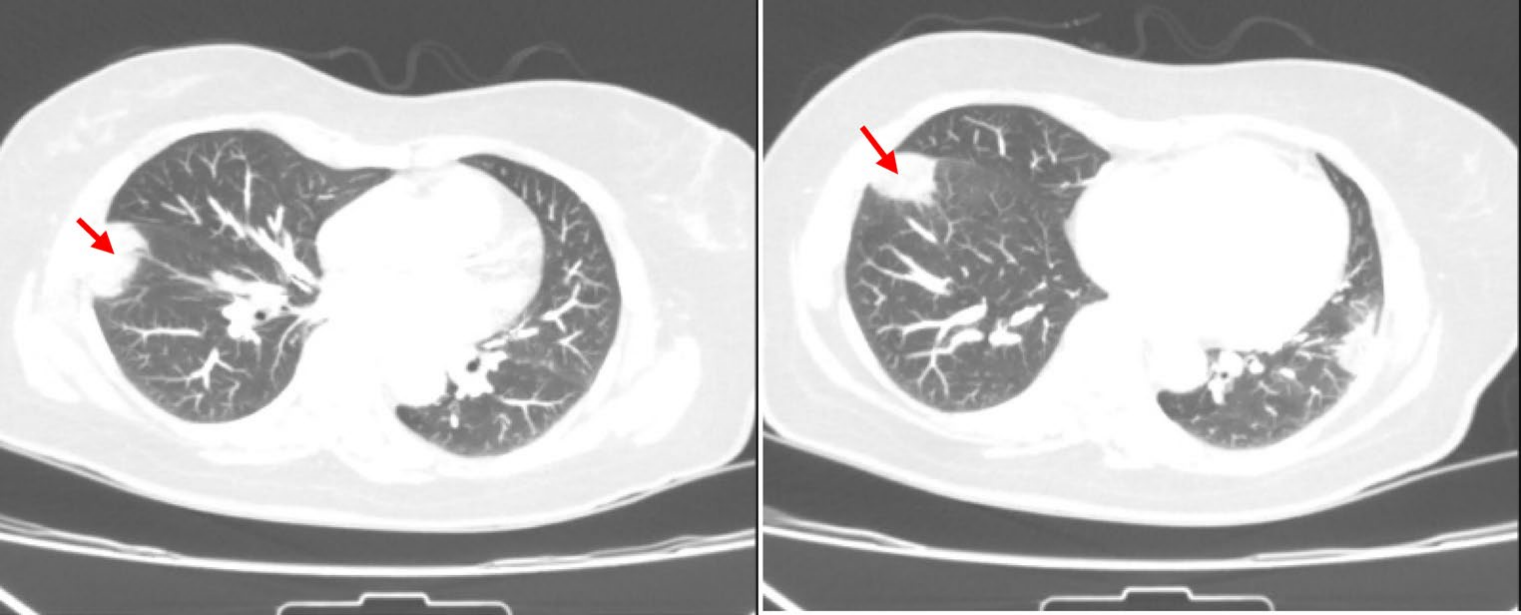
Non-contrast CT showing right lower lobe subpleural wedge-shaped opacity (red arrow) consistent with infarction

**Fig. 2:**
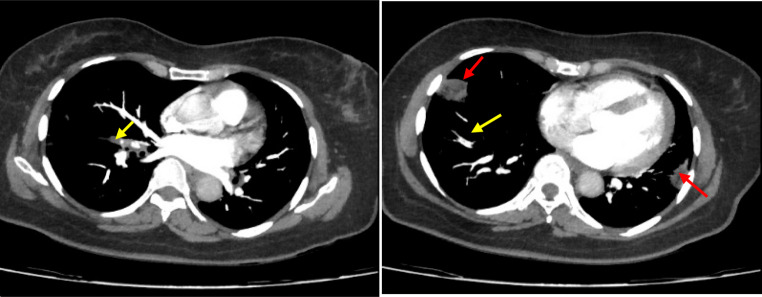
CT pulmonary angiography revealing a segmental filling defect in the right pulmonary artery (yellow arrow) and a subsegmental defect in the left (blue arrow), with associated bilateral infarctions (red arrows)

Chest CT without contrast revealed a right lower lobe wedge-shaped consolidation consistent with infarction (Fig. 1). CT pulmonary angiography confirmed segmental PE within the right lower lobe pulmonary artery and subsegmental PE in the left lower lobe pulmonary artery, with associated infarctions in both lobes (Fig. 2). Echocardiography showed normal biventricular function and an estimated systolic pulmonary artery pressure (sPAP) of 30 mmHg. Bilateral lower extremity Doppler ultrasonography was performed and revealed no evidence of deep vein thrombosis.

A multidisciplinary team (including hematology, pulmonology, and clinical pharmacy) initiated apixaban (with a loading dose of 10 mg twice daily for 7 days, then 5 mg twice daily). Hemoptysis resolved within 48 h. A follow-up echocardiogram at six weeks showed normalization of sPAP (20 mmHg). Given the severity of the provoking factor (major surgery with intense hemostatic support) and the presence of pulmonary infarction, the multidisciplinary team decided on a planned treatment duration of at least 3–6 months, with intent to re-image. However, apixaban was discontinued after four months due to a spontaneous intra-articular bleeding (right knee) causing significant pain and joint swelling. The bleeding event was managed with a single unit of platelet concentrate and one dose of rFVIIa (90 µg/kg), resulting in symptom resolution. The patient experienced no other bleeding events, specifically no exacerbation of her typical mucocutaneous symptoms, during the anticoagulation period. Subsequent cataract surgery three months later was uneventful with appropriate hemostatic cover.

## Discussion and conclusions

This case illustrates the thrombosis-hemorrhage paradox, a significant clinical challenge in the management of congenital bleeding disorders. The development of PE in a patient with GT following a surgical procedure with hemostatic support challenges the notion that hypocoagulability confers absolute protection against thrombotic events. Our patient’s course indicates that acquired prothrombotic stimuli can override this inherent bleeding tendency.

The development of PE in our GT patient is best understood in the context of her specific perioperative management. She received a single, high-dose bolus of rFVIIa (90 µg/kg) as prophylactic coverage for her spinal surgery, in addition to a multi-day course of platelet transfusions. This prophylactic use of rFVIIa, while not standard for all elective procedures in GT, is a clinical practice for high-bleeding-risk surgeries. It is crucial to note that no significant intraoperative bleeding occurred, and the rFVIIa regimen was not continued postoperatively. This suggests that even a single, transient exposure to a high concentration of rFVIIa, when combined with other prothrombotic stimuli like platelet transfusions and the postoperative inflammatory state, can be sufficient to catalyze thrombus formation in susceptible individuals. The potent thrombin-generating capacity of rFVIIa on the surface of the transfused platelets is the most plausible mechanism for this complication [7]. Concurrently, platelet transfusions provide a transient infusion of functional αIIbβ3 integrins, temporarily correcting the fundamental aggregation defect in GT and creating a substrate for rFVIIa to exert its procoagulant effect [6, 8]. This iatrogenic, transient normalization of hemostasis, set against a backdrop of a hypercoagulable postoperative state, appears to have been sufficient to catalyze thrombus formation.

The occurrence of VTE in GT, while rare, is not entirely unexpected from a pathobiological standpoint. As highlighted by Nurden [2], the αIIbβ3 integrin is not the sole player in thrombosis. The related αvβ3 integrin, which is also deficient in many patients with ITGB3 mutations, plays roles in vascular biology, angiogenesis, and inflammation. The absence of these integrins may lead to compensatory mechanisms or alter the vascular microenvironment in ways that do not fully protect against venous thrombosis, particularly when powerful exogenous triggers like rFVIIa are introduced. This broader biological context moves the understanding beyond a simple platelet aggregation defect.

The diagnostic dilemma posed by hemoptysis in this population is considerable. In a GT patient, hemorrhage is the default clinical suspicion. This case underscores the need for clinicians to maintain a high index of suspicion for thrombosis when such patients present with ambiguous symptoms, particularly in the context of recent procedural provocation. Rapid and definitive imaging, specifically computed tomography pulmonary angiography (CTPA), is essential for resolving this diagnostic uncertainty and guiding appropriate management.

The two-month interval between surgery and symptom onset is notably delayed compared to the typical 4–6 week window for post-operative VTE. This suggests that the prothrombotic insult may not be a transient event but rather could induce a sustained hypercoagulable state. While the overall thrombotic risk associated with rFVIIa is low [9], its potent procoagulant effect is well-documented. The delayed presentation raises the possibility of a lasting biological impact from rFVIIa, which may alter vascular homeostasis and promote a prolonged prothrombotic milieu beyond its short circulatory half-life [10]. This effect was likely compounded by a persistent postoperative inflammatory state—a known driver of sustained hypercoagulability that can extend VTE risk for months [11, 12]—and relative immobilization during recovery.

Therapeutic decision-making in this context is fraught with complexity. The selection of apixaban, a direct oral anticoagulant (DOAC), was guided by its favorable pharmacokinetic profile: predictable dose response, short half-life, and minimal requirement for monitoring [13, 14]. These characteristics are particularly advantageous in patients with a high bleeding risk, as they allow for rapid offset of effect in the event of hemorrhage [15]. The findings from our systematic review provide crucial insights into anticoagulant choice. The direct comparison in the case by Sattler et al. [3], where rivaroxaban failed and caused bleeding but apixaban succeeded, strongly supports our choice of a factor Xa inhibitor, suggesting a potentially better safety profile for apixaban in this population.

Our case presents a different scenario compared to Sattler et al. [3]. Our patient had a normal BMI and developed PE following a regimen that included only a single prophylactic dose of rFVIIa, not a continued course. Furthermore, the successful initial use of apixaban followed by a spontaneous hemorrhage at 4 months provides new insight into the time-limited safety of DOACs in GT, suggesting a maximum safe duration may be around 3 months.

While other agents like low-molecular-weight heparin (LMWH) or dabigatran offer specific reversal strategies (protamine and idarucizumab, respectively), the patient’s strong preference for an oral agent was a deciding factor. It is crucial to note that andexanet al.fa for factor Xa inhibitor reversal was unavailable at our institution, a limitation that must be factored into anticoagulant selection at other centers; in such scenarios, prothrombin complex concentrate (PCC) can be considered as a non-specific reversal agent. The successful resolution of the PE without initial hemorrhagic complication adds to the growing, albeit limited, body of evidence supporting the short-term use of DOACs in complex bleeding disorders [16–18].

However, the subsequent spontaneous intra-articular hemorrhage after four months of successful therapy serves as a clear reminder of the narrow therapeutic window in GT. This event indicates that while intermediate-term DOAC therapy can be effective and safe, the relentless baseline bleeding diathesis ultimately prevails, mandating a finite treatment duration. The decision to treat for four months was based on the high-risk provoking factor, but the bleeding complication confirms that extending therapy beyond 3 months in such patients requires extreme caution and a clear reassessment of the risk-benefit ratio. This outcome is consistent with findings from our systematic review (Table 2), which reveals that bleeding complications on anticoagulation are not uncommon in this population, yet successful outcomes are frequently achieved with careful management.

**Table 2:** Systematic review of thrombotic events and management in Glanzmann Thrombasthenia

Reference (Year)	Age/Sex	GT Type/Diagnosis Method (CD41/CD61% if available)	Thrombotic Event Type & Location	Provoking/Risk Factors	Anticoagulant Management (Drug, Dose, Duration)	Outcome (Thrombosis)	Bleeding Complications on AC	Other Management Notes
Sattler et al. (2023) [3]	40/F	Type I (Flow Cytometry, Genetic)	Recurrent DVT (Popliteal vein).	- rFVIIa use. - Morbid Obesity. - Smoking. - Family History of VTE.	Rivaroxaban → Apixaban for 18 months cumulative.	Failure on Rivaroxaban; Success on Apixaban.	Menometrorrhagia on Rivaroxaban; None on Apixaban.	Direct evidence for the superior safety/efficacy of Apixaban over Rivaroxaban in this population.
Schutgens et al. (2021) [4]	74/F	Type I (Genetic)	Recurrent DVT & Superficial Thrombophlebitis.	- JAK2 + Polycythemia Vera. - Age. - Hypertension. - Smoking History.	Dabigatran (titrated up to 150 mg BID) + Hydroxyurea.	Failure on low-dose; Success on high-dose + cytoreduction.	None reported.	Concomitant MPN can overpower the GT defect, requiring aggressive, multi-modal antithrombotic therapy.
Giuffrida et al. (2017) [19] (Patient 1)	37/M	Type I (Flow Cytometry, Genetic: <1% CD41/CD61)	DVT (Tibial posterior & Popliteal vein).	- Physical exercise. - Negative thrombophilia.	LMWH (80 IU/kg OD → 100 IU/kg BID) for 7 months.	Failure on prophylactic dose; Success on therapeutic dose.	No severe haemorrhages.	Critical demonstration that full therapeutic-dose LMWH is required for efficacy.
Giuffrida et al. (2017) [19] (Patient 2)	77/M	Type I (Flow Cytometry, Genetic: <1% CD41/CD61)	Atrial Fibrillation (Stroke prevention).	- Age. - Atrial Fibrillation.	LMWH (50 IU/kg BID) for 13 + months.	N/A (Primary prevention). Successful.	No severe bleeding. (Ecchymoses).	Long-term adjusted-dose LMWH is viable for chronic anticoagulation in GT.
Rezende (2011) [20]	36/M	Type I (Aggregometry, Flow Cytometry)	Recurrent DVT.	- Heterozygous FVL. - Positive LA. - Strong Family History.	Low-Intensity Warfarin (INR 1.5–2.0.5.0) long-term.	Success.	Minor bleeding only.	Low-intensity VKA is safe and effective for long-term secondary prevention.
Phillips et al. (2007) [5]	2/F	Type I (Aggregometry, Flow Cytometry)	Femoral DVT (catheter-related).	- Central Venous Catheter.	None (catheter removed).	Resolution.	N/A.	Conservative management without AC can be valid for transient, removable triggers in high-bleeding-risk settings.
Ten Cate et al. (2003) [21]	48/M	Type I Dx Method: "Glanzmann’s type 1 disease”. CD41/CD61: Not reported.	Recurrent Proximal DVT (x3).	- Heterozygous FVL. - Smoking. - Immobilization.	Long-term/maintenance LMWH (≥ 4 years).	Success.	Yes. Trauma-related hematoma.	Early case establishing the need for long-term prophylaxis with LMWH.
Gruel et al. (1997) [22]	67/M	Variant (~ 50% defective CD41/CD61)	Extensive Proximal DVT.	- Prolonged immobilization (flight/car). - Age.	LMWH (Dalteparin) for 6 weeks (monitored).	Success.	None.	Pioneering case. Proved VTE can occur in GT and that monitored LMWH is a safe and effective treatment.

*Abbreviations*: *AC* Anticoagulation, *ADP* Adenosine Diphosphate, *AF* Atrial Fibrillation, *BID* Twice Daily, *CTPA* Computed Tomography Pulmonary Angiography, *DOAC* Direct Oral Anticoagulant, *DVT* Deep Vein Thrombosis, *FVL* Factor V Leiden, *GP* Glycoprotein, *GT* Glanzmann Thrombasthenia, *JAK2* Janus Kinase 2, *LA* Lupus Anticoagulant, *LMWH* Low-Molecular-Weight Heparin, *LTA* Light Transmission Aggregometry, *MPN* Myeloproliferative Neoplasm, *OD* Once Daily, *PCC* Prothrombin Complex Concentrate, *PE* Pulmonary Embolism, *rFVIIa* Recombinant Factor VIIa, *sPAP* Systolic Pulmonary Artery Pressure, *VKA* Vitamin K Antagonist, *VTE* Venous Thromboembolism

### Clinical implications and future directions

This case suggests the need to re-evaluate passive approaches to thromboprophylaxis in GT. The historical imperative to avoid bleeding may have led to the systematic under-prophylaxis of high-risk patients.

We propose that a mandatory baseline of aggressive mechanical prophylaxis (e.g., intermittent pneumatic compression) should be implemented for all GT patients undergoing major surgery or facing immobilization. The decision to escalate to pharmacologic prophylaxis must be multidisciplinary, involving hematologists, surgeons, and pharmacists. It may be justified in the highest-risk scenarios for a short, defined perioperative period, with extreme vigilance for bleeding. Our literature review suggests that adjusted-dose LMWH may be a viable option for this purpose [19], but this decision must be highly individualized. Furthermore, the risk associated with rFVIIa must be carefully considered; its use should be meticulously weighed, and the minimum effective dose and duration should be employed.

This case confirms that Glanzmann Thrombasthenia does not guarantee protection from thrombosis. In our patient, a single surgical episode with hemostatic support was enough to provoke a pulmonary embolism. Apixaban provided effective short-term treatment, but the hemorrhage that occurred months later was an inevitable reminder of the underlying bleeding risk. This delayed complication suggests a practical limit for anticoagulation—around three months—beyond which the danger of bleeding likely outweighs the benefit. Navigating this narrow window remains a central challenge in managing thrombosis in these complex patients.

## Supplementary Information

Below is the link to the electronic supplementary material.

Supplementary Material 1

## Data Availability

The data that support the findings of this study are available from the corresponding author, [N.K], upon reasonable request.

## Declarations

### Artificial intelligence assistance

During the preparation of this work, the authors used DeepSeek AI solely for the purpose of improving language, readability, and grammar. After using this tool, the authors reviewed and edited the content as needed and take full responsibility for the final version of the manuscript.

## Abbreviations

ADP: Adenosine Diphosphate
AF: Atrial Fibrillation
CTPA: Computed Tomography Pulmonary Angiography
DOAC: Direct Oral Anticoagulant
DVT: Deep Vein Thrombosis
GP: Glycoprotein
GT: Glanzmann Thrombasthenia
JAK2: Janus Kinase 2
LMWH: Low-Molecular-Weight Heparin
LTA: Light Transmission Aggregometry
MPN: Myeloproliferative Neoplasm
PE: Pulmonary Embolism
rFVIIa: Recombinant Factor VIIa
sPAP: Systolic Pulmonary Artery Pressure
VTE: Venous Thromboembolism
AC: Anticoagulation
BID: Twice Daily
FVL: Factor V Leiden
LA: Lupus Anticoagulant
OD: Once Daily
PCC: Prothrombin Complex Concentrate
VKA: Vitamin K Antagonist
VTE: Venous Thromboembolism
