# Supplementary material for: Pulmonary thromboembolism in Glanzmann Thrombasthenia: a case report and systematic literature review
Source: Ann Hematol. 2026 Jan 27;105(2):64. doi: 10.1007/s00277-026-06732-8 (PMC12847078; doi:10.1007/s00277-026-06732-8)
Supplement: Supplementary file 1 — Supplementary Material 1 [file 277_2026_6732_MOESM1_ESM.docx]

**Search Strategy**

A systematic literature search was performed on August 12, 2024, in the following databases from inception:

**PubMed:**

("Thrombasthenia"[Mesh] OR "Glanzmann Thrombasthenia"[tiab] OR "Glanzmann's Thrombasthenia"[tiab] OR "platelet function disorder"[tiab] OR "GPIIbIIIa deficiency"[tiab] OR "GP IIb IIIa deficiency"[tiab]) AND ("Thrombosis"[Mesh] OR "Thromboembolism"[Mesh] OR "Venous Thromboembolism"[Mesh] OR thrombosis[tiab] OR thromboembolism[tiab] OR "pulmonary embolism"[tiab] OR "deep vein thrombosis"[tiab] OR "venous thrombosis"[tiab])

**Scopus:**

( TITLE-ABS-KEY ( "Glanzmann Thrombasthenia" OR "Glanzmann's Thrombasthenia" OR "Thrombasthenia" OR "platelet function disorder" OR "GPIIbIIIa deficiency" OR "GP IIb IIIa deficiency" ) ) AND ( TITLE-ABS-KEY ( thrombosis OR thromboembolism OR "pulmonary embolism" OR "deep vein thrombosis" OR "venous thrombosis" ) )

*No filters or limits were applied to the search.*

**
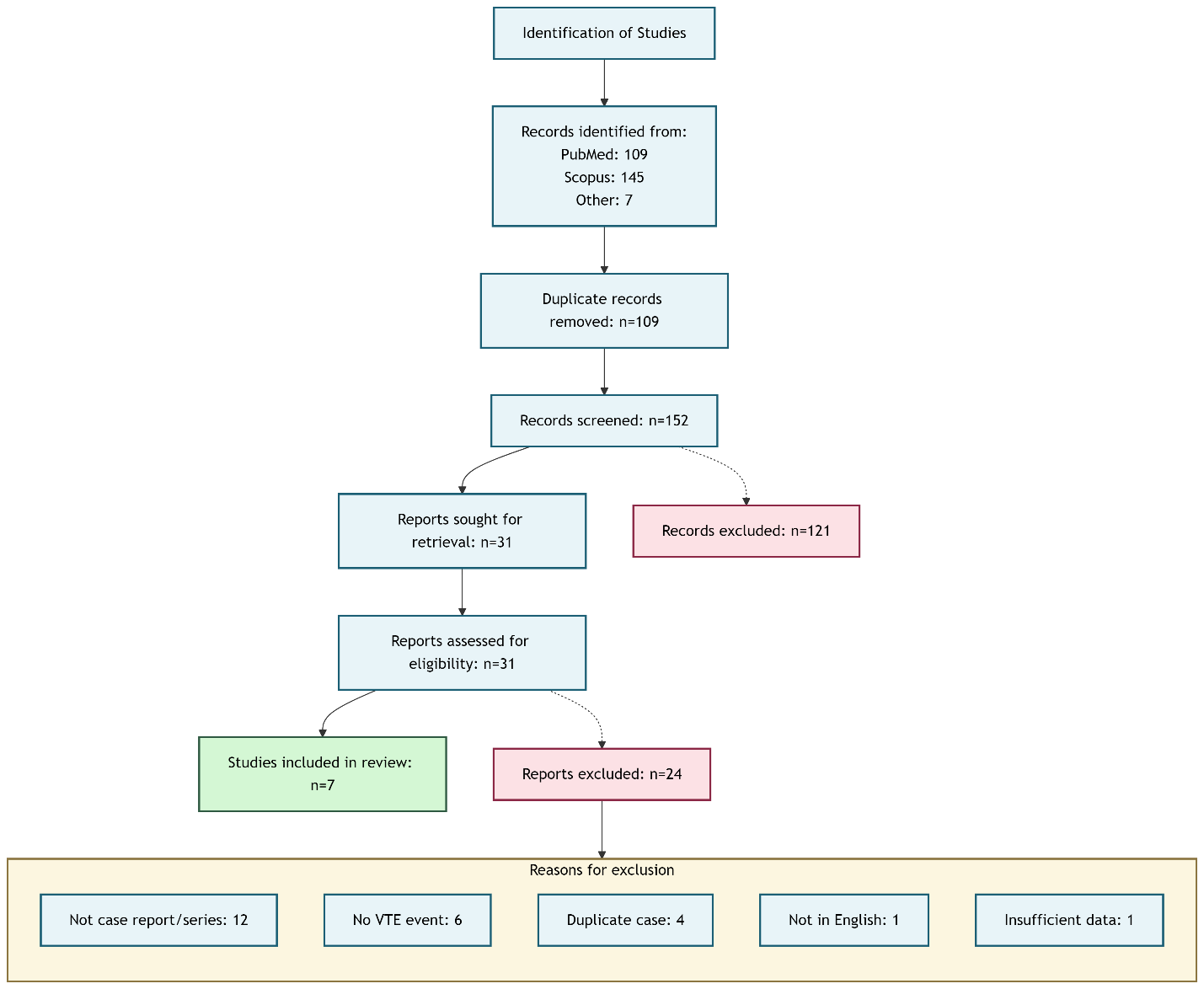
**

**PRISMA Flow Diagram**
